# Methyl 1-methyl-3-*p*-tolyl-1,2,3,3a,4,11c-hexa­hydro­benzo[*f*]chromeno[4,3-*b*]pyrrole-3a-carboxyl­ate

**DOI:** 10.1107/S1600536809024738

**Published:** 2009-07-11

**Authors:** S. Nirmala, E. Theboral Sugi Kamala, L. Sudha, S. Kathiravan, R. Raghunathan

**Affiliations:** a Department of Physics, Easwari Engineering College, Ramapuram, Chennai 600 089, India; b Department of Physics, SRM University, Ramapuram Campus, Chennai 600 089, India; c Department of Organic Chemistry, University of Madras, Guindy Campus, Chennai 600 025, India

## Abstract

In the title compound, C_25_H_25_NO_3_, the dihydro­pyran ring adopts a half-chair conformation, whereas the pyrrolidine ring is in a twist conformation. The tolyl group is oriented at an angle of 82.92 (7)° with respect to the napthalene ring system. In the crystal structure, mol­ecules are linked into centrosymmetric dimers by C—H⋯π inter­actions involving the benzene ring of the tolyl group.

## Related literature

For the biological activity of pyrrole derivatives, see: Biava *et al.* (2005 ▶); Borthwick *et al.* (2000 ▶); Caine (1993 ▶); Carlson (1993 ▶); Fernandes *et al.* (2004 ▶); Jiang *et al.* (2004 ▶); Sokoloff *et al.* (1990 ▶); Tidey & Miczek (1992 ▶); Wilner (1985 ▶). For a related structure, see: Gunasekaran *et al.* (2009 ▶). For ring-puckering parameters, see: Cremer & Pople (1975 ▶).
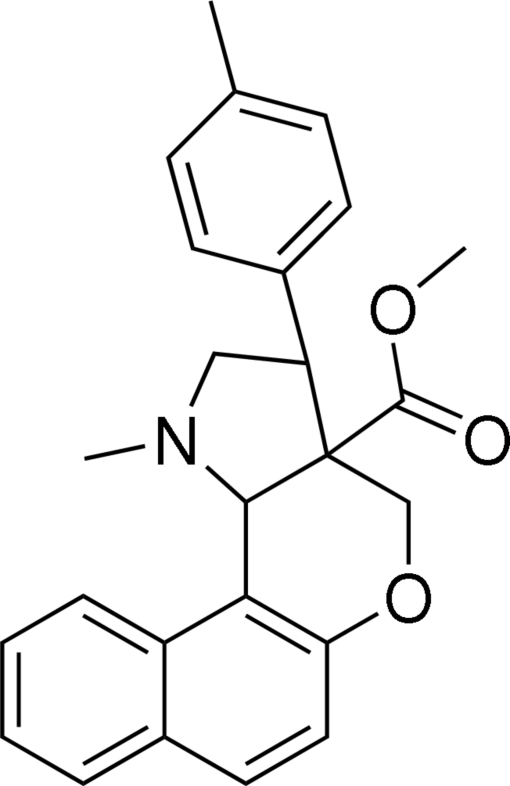


## Experimental

### 

#### Crystal data

C_25_H_25_NO_3_
*M* *_r_* = 387.46
Monoclinic, 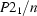

*a* = 12.9899 (6) Å
*b* = 7.6751 (3) Å
*c* = 20.5073 (9) Å
β = 96.881 (2)°
*V* = 2029.83 (15) Å^3^
*Z* = 4
Mo *K*α radiation
μ = 0.08 mm^−1^
*T* = 293 K
0.25 × 0.20 × 0.20 mm

#### Data collection

Bruker Kappa-APEXII area-detector diffractometer
Absorption correction: multi-scan (Blessing, 1995 ▶) *T*_min_ = 0.980, *T*_max_ = 0.984
22975 measured reflections
4790 independent reflections
3176 reflections with *I* > 2σ(*I*)
*R*_int_ = 0.028

#### Refinement

*R*[*F* ^2^ > 2σ(*F* ^2^)] = 0.047
*wR*(*F* ^2^) = 0.156
*S* = 1.05
4790 reflections
265 parameters
H-atom parameters constrained
Δρ_max_ = 0.24 e Å^−3^
Δρ_min_ = −0.19 e Å^−3^

### 

Data collection: *APEX2* (Bruker, 2004 ▶); cell refinement: *SAINT* (Bruker, 2004 ▶); data reduction: *SAINT*; program(s) used to solve structure: *SHELXS97* (Sheldrick, 2008 ▶); program(s) used to refine structure: *SHELXL97* (Sheldrick, 2008 ▶); molecular graphics: *ORTEP-3* (Farrugia, 1997 ▶); software used to prepare material for publication: *PLATON* (Spek, 2009 ▶).

## Supplementary Material

Crystal structure: contains datablocks I, global. DOI: 10.1107/S1600536809024738/ci2823sup1.cif

Structure factors: contains datablocks I. DOI: 10.1107/S1600536809024738/ci2823Isup2.hkl

Additional supplementary materials:  crystallographic information; 3D view; checkCIF report

## Figures and Tables

**Table 1:** Hydrogen-bond geometry (Å, °)

*D*—H⋯*A*	*D*—H	H⋯*A*	*D*⋯*A*	*D*—H⋯*A*
C22—H22*A*⋯*Cg*1^i^	0.96	2.84	3.788 (2)	169

Symmetry code: (i) 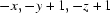. *Cg*1 is the centroid of the C16–C21 ring.

**Fig. 1.**
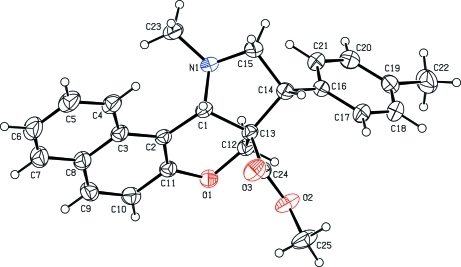
The molecular structure of the title compound, with 30% probability displacement ellipsoids.

**Fig. 2.**
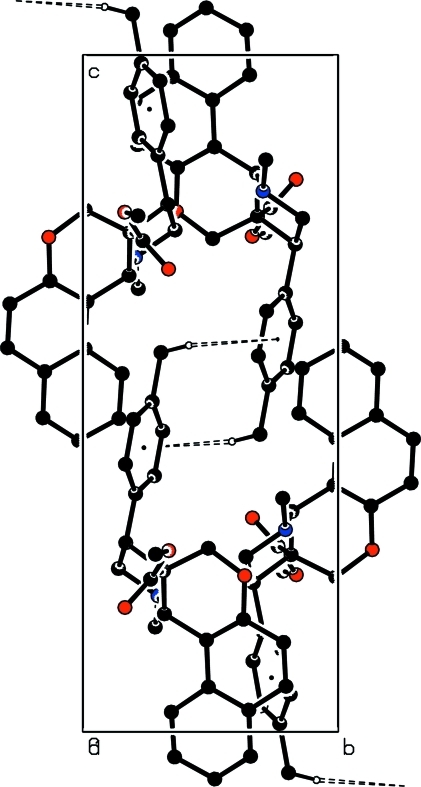
The packing of the molecules viewed down the a axis. H atoms not involed in C—H···π interactions (dashed lines) have been omitted.

## supplementary crystallographic information

### Comment

Chromenopyrrole compounds are used in the treatment of impulsive disorders (Caine, 1993), aggressiveness (Tidey & Miczek, 1992), parkinson's disease (Carlson, 1993), psychoses, memory disorders (Sokoloff *et al.*, 1990), anxiety and depression (Wilner, 1985). Pyrrole derivatives have good in vitro activities against mycobacteria and candidae (Biava *et al.*, 2005). These derivatives also possess anti-inflammatory (Fernandes *et al.*, 2004) and antiviral (Borthwick *et al.*, 2000) activities. It has also been shown that N–substituted pyrrole derivatives inhibit human immuno deficiency virus type-I (HIV-I) (Jiang *et al.*, 2004). In view of its medicinal importance, the crystal structure determination of the title compound was undertaken.

The geometric parameters of the title molecule (Fig. 1) agree well with those reported for a similar structure (Gunasekaran *et al.*, 2009). The sum of bond angles around atom N1 (334.0°) is in accordance with *sp*^3^ hybridization. The napthalene ring system (C2-C11) and the tolyl group (C16-C22) are oriented at an angle of 82.92 (7)° with respect to each other. The heterocyclic ring (O1/C1/C2/C11-C13) of the chromenopyrrole unit adopts a half-chair conformation, with puckering parameters *Q* = 0.462 (2) Å, *θ* = 49.3 (2)° and *φ*= 261.2 (2)° (Cremer and Pople, 1975). The pyrrolidine ring (N1/C1/C13-C15) adopts a twist conformation, with puckering parameters of *q*_2_ = 0.485 (1) Å and *φ* = 16.2 (2)° (Cremer and Pople, 1975).

The crystal packing is stabilized by weak intermolecular C—H···π [C22—H22A···*Cg1*; *Cg1* is the centroid of the C16—C21 ring] interactions (Table 1).

### Experimental

A mixture of (*Z*)-methyl 2-((1-formylnaphthalen-2-yloxy)methyl)-3- *p*-tolylacrylate (20 mmol) and sarcosine (30 mmol) was refluxed in benzene for 20 h and the solvent was removed under reduced pressure. The crude product was subjected to column chromatography to get the pure product. A chloroform and methanol (1:1) solvent mixture was used for the crystallization under slow evaporation method.

### Refinement

H atoms were placed in idealized positions and allowed to ride on their parent atoms, with C-H = 0.93, 0.98, 0.97 and 0.96 Å for aromatic, methine, methylene and methyl H respectively, and *U*_iso_(H) = 1.5U_eq_(C) for methyl H and *U*_iso_(H) = 1.2U_eq_(C) for all other H atoms.

### Crystal data

**Table:**

C_25_H_25_NO_3_	*F*(000) = 824
*M**_r_* = 387.46	*D*_x_ = 1.268 Mg m^−^^3^
Monoclinic, *P*2_1_/*n*	Mo *K*α radiation, λ = 0.71073 Å
Hall symbol: -P 2yn	Cell parameters from 6463 reflections
*a* = 12.9899 (6) Å	θ = 2.8–25.6°
*b* = 7.6751 (3) Å	µ = 0.08 mm^−^^1^
*c* = 20.5073 (9) Å	*T* = 293 K
β = 96.881 (2)°	Prism, colourless
*V* = 2029.83 (15) Å^3^	0.25 × 0.20 × 0.20 mm
*Z* = 4	

### Data collection

**Table:**

Bruker Kappa-APEXII area-detector diffractometer	4790 independent reflections
Radiation source: fine-focus sealed tube	3176 reflections with *I* > 2σ(*I*)
graphite	*R*_int_ = 0.028
ω and φ scans	θ_max_ = 27.8°, θ_min_ = 1.8°
Absorption correction: multi-scan (Blessing, 1995)	*h* = −17→16
*T*_min_ = 0.980, *T*_max_ = 0.984	*k* = −8→10
22975 measured reflections	*l* = −20→26

### Refinement

**Table:**

Refinement on *F*^2^	Primary atom site location: structure-invariant direct methods
Least-squares matrix: full	Secondary atom site location: difference Fourier map
*R*[*F*^2^ > 2σ(*F*^2^)] = 0.047	Hydrogen site location: inferred from neighbouring sites
*wR*(*F*^2^) = 0.156	H-atom parameters constrained
*S* = 1.05	*w* = 1/[σ^2^(*F*_o_^2^) + (0.081*P*)^2^ + 0.2107*P*] where *P* = (*F*_o_^2^ + 2*F*_c_^2^)/3
4790 reflections	(Δ/σ)_max_ = 0.001
265 parameters	Δρ_max_ = 0.24 e Å^−^^3^
0 restraints	Δρ_min_ = −0.19 e Å^−^^3^

### Special details

**Table:**

Geometry. All e.s.d.'s (except the e.s.d. in the dihedral angle between two l.s. planes) are estimated using the full covariance matrix. The cell e.s.d.'s are taken into account individually in the estimation of e.s.d.'s in distances, angles and torsion angles; correlations between e.s.d.'s in cell parameters are only used when they are defined by crystal symmetry. An approximate (isotropic) treatment of cell e.s.d.'s is used for estimating e.s.d.'s involving l.s. planes.
Refinement. Refinement of *F*^2^ against ALL reflections. The weighted *R*-factor *wR* and goodness of fit *S* are based on *F*^2^, conventional *R*-factors *R* are based on *F*, with *F* set to zero for negative *F*^2^. The threshold expression of *F*^2^ > σ(*F*^2^) is used only for calculating *R*-factors(gt) *etc*. and is not relevant to the choice of reflections for refinement. *R*-factors based on *F*^2^ are statistically about twice as large as those based on *F*, and *R*- factors based on ALL data will be even larger.

### Fractional atomic coordinates and isotropic or equivalent isotropic displacement parameters (Å^2^)

**Table:**

	*x*	*y*	*z*	*U*_iso_*/*U*_eq_	
C1	−0.09259 (11)	0.31953 (19)	0.17432 (7)	0.0388 (3)	
H1	−0.0790	0.2192	0.1471	0.047*	
C2	−0.08470 (10)	0.48434 (19)	0.13573 (7)	0.0384 (3)	
C3	−0.11550 (11)	0.4919 (2)	0.06634 (7)	0.0446 (4)	
C4	−0.15523 (13)	0.3466 (3)	0.02933 (8)	0.0558 (4)	
H4	−0.1621	0.2406	0.0503	0.067*	
C5	−0.18383 (15)	0.3592 (3)	−0.03703 (9)	0.0697 (6)	
H5	−0.2104	0.2622	−0.0605	0.084*	
C6	−0.17327 (16)	0.5168 (4)	−0.06962 (10)	0.0765 (6)	
H6	−0.1929	0.5245	−0.1147	0.092*	
C7	−0.13492 (15)	0.6571 (3)	−0.03608 (9)	0.0681 (6)	
H7	−0.1283	0.7612	−0.0584	0.082*	
C8	−0.10438 (12)	0.6502 (2)	0.03225 (8)	0.0527 (4)	
C9	−0.06138 (14)	0.7959 (2)	0.06742 (9)	0.0569 (5)	
H9	−0.0551	0.9007	0.0454	0.068*	
C10	−0.02925 (12)	0.7865 (2)	0.13232 (9)	0.0499 (4)	
H10	−0.0001	0.8835	0.1546	0.060*	
C11	−0.04003 (11)	0.6293 (2)	0.16629 (7)	0.0412 (4)	
C12	−0.02357 (12)	0.4915 (2)	0.27124 (7)	0.0421 (4)	
H12A	−0.0931	0.5077	0.2830	0.051*	
H12B	0.0241	0.4923	0.3115	0.051*	
C13	−0.01754 (11)	0.31594 (19)	0.23809 (7)	0.0383 (3)	
C14	−0.06451 (12)	0.1678 (2)	0.27941 (8)	0.0460 (4)	
H14	−0.0239	0.0616	0.2752	0.055*	
C15	−0.17243 (13)	0.1390 (2)	0.24138 (8)	0.0541 (4)	
H15A	−0.1732	0.0353	0.2143	0.065*	
H15B	−0.2245	0.1268	0.2713	0.065*	
C16	−0.06274 (12)	0.2062 (2)	0.35187 (8)	0.0452 (4)	
C17	0.02402 (13)	0.1666 (2)	0.39539 (9)	0.0560 (5)	
H17	0.0815	0.1176	0.3794	0.067*	
C18	0.02723 (15)	0.1980 (3)	0.46167 (10)	0.0649 (5)	
H18	0.0867	0.1691	0.4895	0.078*	
C19	−0.05580 (15)	0.2713 (2)	0.48788 (8)	0.0584 (5)	
C20	−0.14203 (14)	0.3124 (2)	0.44449 (9)	0.0579 (5)	
H20	−0.1991	0.3631	0.4604	0.070*	
C21	−0.14554 (13)	0.2801 (2)	0.37841 (9)	0.0537 (4)	
H21	−0.2052	0.3086	0.3507	0.064*	
C22	−0.0533 (2)	0.3059 (3)	0.56044 (9)	0.0838 (7)	
H22A	−0.0196	0.4154	0.5711	0.126*	
H22B	−0.0157	0.2144	0.5848	0.126*	
H22C	−0.1229	0.3100	0.5717	0.126*	
C23	−0.28437 (12)	0.2833 (2)	0.15488 (9)	0.0548 (4)	
H23A	−0.2814	0.1813	0.1281	0.082*	
H23B	−0.2897	0.3850	0.1275	0.082*	
H23C	−0.3438	0.2763	0.1784	0.082*	
C24	0.09063 (12)	0.2649 (2)	0.22557 (8)	0.0416 (4)	
C25	0.26911 (14)	0.2845 (3)	0.26242 (13)	0.0781 (6)	
H25A	0.2773	0.1627	0.2723	0.117*	
H25B	0.3156	0.3507	0.2929	0.117*	
H25C	0.2847	0.3057	0.2185	0.117*	
N1	−0.19140 (9)	0.29402 (17)	0.20102 (6)	0.0447 (3)	
O1	0.00093 (8)	0.63413 (14)	0.23080 (5)	0.0468 (3)	
O2	0.16331 (8)	0.33635 (16)	0.26780 (7)	0.0646 (4)	
O3	0.10931 (9)	0.16459 (17)	0.18379 (6)	0.0630 (4)	

### Atomic displacement parameters (Å^2^)

**Table:**

	*U*^11^	*U*^22^	*U*^33^	*U*^12^	*U*^13^	*U*^23^
C1	0.0344 (7)	0.0380 (8)	0.0430 (8)	−0.0011 (6)	0.0001 (6)	−0.0058 (7)
C2	0.0304 (7)	0.0415 (8)	0.0431 (8)	0.0015 (6)	0.0038 (6)	0.0003 (7)
C3	0.0321 (7)	0.0576 (10)	0.0441 (8)	0.0046 (7)	0.0051 (6)	−0.0006 (8)
C4	0.0458 (9)	0.0734 (12)	0.0471 (9)	−0.0008 (8)	0.0007 (7)	−0.0069 (9)
C5	0.0537 (11)	0.1041 (17)	0.0497 (10)	−0.0019 (10)	−0.0004 (8)	−0.0152 (12)
C6	0.0628 (12)	0.123 (2)	0.0424 (10)	0.0045 (12)	0.0015 (9)	0.0075 (13)
C7	0.0589 (11)	0.0952 (16)	0.0508 (11)	0.0062 (11)	0.0099 (9)	0.0179 (11)
C8	0.0393 (8)	0.0695 (12)	0.0498 (9)	0.0082 (8)	0.0079 (7)	0.0100 (9)
C9	0.0514 (10)	0.0550 (10)	0.0657 (12)	0.0057 (8)	0.0125 (9)	0.0172 (9)
C10	0.0451 (9)	0.0411 (9)	0.0641 (11)	0.0012 (7)	0.0087 (8)	0.0018 (8)
C11	0.0346 (7)	0.0417 (8)	0.0475 (9)	0.0029 (6)	0.0060 (6)	0.0003 (7)
C12	0.0416 (8)	0.0425 (8)	0.0412 (8)	−0.0030 (6)	0.0009 (6)	−0.0026 (7)
C13	0.0356 (7)	0.0374 (8)	0.0405 (8)	−0.0027 (6)	−0.0004 (6)	−0.0008 (7)
C14	0.0456 (9)	0.0403 (8)	0.0514 (9)	−0.0048 (6)	0.0026 (7)	0.0029 (7)
C15	0.0522 (10)	0.0549 (10)	0.0532 (10)	−0.0157 (8)	−0.0020 (8)	0.0042 (8)
C16	0.0408 (8)	0.0444 (9)	0.0492 (9)	−0.0051 (7)	0.0006 (7)	0.0091 (7)
C17	0.0427 (9)	0.0659 (11)	0.0578 (11)	0.0057 (8)	0.0004 (8)	0.0116 (9)
C18	0.0535 (11)	0.0802 (13)	0.0570 (11)	−0.0022 (9)	−0.0100 (8)	0.0153 (10)
C19	0.0630 (11)	0.0621 (11)	0.0489 (10)	−0.0158 (9)	0.0020 (9)	0.0107 (9)
C20	0.0515 (10)	0.0659 (11)	0.0578 (11)	−0.0034 (8)	0.0123 (8)	0.0048 (9)
C21	0.0420 (9)	0.0638 (11)	0.0535 (10)	0.0009 (8)	−0.0013 (7)	0.0090 (9)
C22	0.1037 (18)	0.0954 (17)	0.0512 (11)	−0.0230 (14)	0.0053 (11)	0.0049 (11)
C23	0.0395 (9)	0.0615 (11)	0.0613 (10)	−0.0084 (7)	−0.0022 (8)	−0.0030 (9)
C24	0.0399 (8)	0.0368 (8)	0.0468 (9)	−0.0003 (6)	−0.0005 (7)	0.0044 (7)
C25	0.0356 (10)	0.0670 (13)	0.1270 (19)	0.0046 (8)	−0.0098 (10)	−0.0069 (12)
N1	0.0348 (7)	0.0491 (8)	0.0489 (7)	−0.0082 (5)	−0.0005 (5)	−0.0006 (6)
O1	0.0517 (6)	0.0394 (6)	0.0475 (6)	−0.0069 (5)	−0.0009 (5)	−0.0037 (5)
O2	0.0366 (6)	0.0623 (8)	0.0905 (9)	0.0003 (5)	−0.0102 (6)	−0.0199 (7)
O3	0.0515 (7)	0.0710 (8)	0.0657 (8)	0.0128 (6)	0.0039 (6)	−0.0153 (7)

### Geometric parameters (Å, °)

**Table:**

C1—N1	1.4676 (18)	C14—C15	1.536 (2)
C1—C2	1.502 (2)	C14—H14	0.98
C1—C13	1.5348 (19)	C15—N1	1.454 (2)
C1—H1	0.98	C15—H15A	0.97
C2—C11	1.371 (2)	C15—H15B	0.97
C2—C3	1.432 (2)	C16—C21	1.384 (2)
C3—C4	1.411 (2)	C16—C17	1.385 (2)
C3—C8	1.418 (2)	C17—C18	1.376 (3)
C4—C5	1.370 (2)	C17—H17	0.93
C4—H4	0.93	C18—C19	1.381 (3)
C5—C6	1.397 (3)	C18—H18	0.93
C5—H5	0.93	C19—C20	1.381 (3)
C6—C7	1.341 (3)	C19—C22	1.508 (3)
C6—H6	0.93	C20—C21	1.373 (2)
C7—C8	1.411 (3)	C20—H20	0.93
C7—H7	0.93	C21—H21	0.93
C8—C9	1.409 (3)	C22—H22A	0.96
C9—C10	1.348 (2)	C22—H22B	0.96
C9—H9	0.93	C22—H22C	0.96
C10—C11	1.409 (2)	C23—N1	1.4445 (19)
C10—H10	0.93	C23—H23A	0.96
C11—O1	1.3661 (18)	C23—H23B	0.96
C12—O1	1.4318 (18)	C23—H23C	0.96
C12—C13	1.516 (2)	C24—O3	1.1983 (19)
C12—H12A	0.97	C24—O2	1.3214 (19)
C12—H12B	0.97	C25—O2	1.448 (2)
C13—C24	1.510 (2)	C25—H25A	0.96
C13—C14	1.583 (2)	C25—H25B	0.96
C14—C16	1.512 (2)	C25—H25C	0.96
			
N1—C1—C2	115.26 (12)	C15—C14—H14	107.7
N1—C1—C13	100.04 (11)	C13—C14—H14	107.7
C2—C1—C13	112.81 (12)	N1—C15—C14	104.68 (12)
N1—C1—H1	109.4	N1—C15—H15A	110.8
C2—C1—H1	109.4	C14—C15—H15A	110.8
C13—C1—H1	109.4	N1—C15—H15B	110.8
C11—C2—C3	118.29 (14)	C14—C15—H15B	110.8
C11—C2—C1	119.61 (13)	H15A—C15—H15B	108.9
C3—C2—C1	121.93 (13)	C21—C16—C17	116.58 (15)
C4—C3—C8	117.66 (15)	C21—C16—C14	123.05 (14)
C4—C3—C2	122.89 (15)	C17—C16—C14	120.37 (15)
C8—C3—C2	119.44 (15)	C18—C17—C16	121.60 (17)
C5—C4—C3	121.10 (19)	C18—C17—H17	119.2
C5—C4—H4	119.5	C16—C17—H17	119.2
C3—C4—H4	119.5	C17—C18—C19	121.54 (17)
C4—C5—C6	120.4 (2)	C17—C18—H18	119.2
C4—C5—H5	119.8	C19—C18—H18	119.2
C6—C5—H5	119.8	C20—C19—C18	116.95 (17)
C7—C6—C5	120.10 (18)	C20—C19—C22	121.04 (19)
C7—C6—H6	119.9	C18—C19—C22	122.01 (19)
C5—C6—H6	119.9	C21—C20—C19	121.54 (17)
C6—C7—C8	121.5 (2)	C21—C20—H20	119.2
C6—C7—H7	119.2	C19—C20—H20	119.2
C8—C7—H7	119.2	C20—C21—C16	121.79 (16)
C9—C8—C7	121.71 (18)	C20—C21—H21	119.1
C9—C8—C3	119.04 (15)	C16—C21—H21	119.1
C7—C8—C3	119.24 (18)	C19—C22—H22A	109.5
C10—C9—C8	121.28 (16)	C19—C22—H22B	109.5
C10—C9—H9	119.4	H22A—C22—H22B	109.5
C8—C9—H9	119.4	C19—C22—H22C	109.5
C9—C10—C11	119.79 (16)	H22A—C22—H22C	109.5
C9—C10—H10	120.1	H22B—C22—H22C	109.5
C11—C10—H10	120.1	N1—C23—H23A	109.5
O1—C11—C2	124.01 (13)	N1—C23—H23B	109.5
O1—C11—C10	113.90 (13)	H23A—C23—H23B	109.5
C2—C11—C10	122.05 (14)	N1—C23—H23C	109.5
O1—C12—C13	113.13 (11)	H23A—C23—H23C	109.5
O1—C12—H12A	109.0	H23B—C23—H23C	109.5
C13—C12—H12A	109.0	O3—C24—O2	123.05 (15)
O1—C12—H12B	109.0	O3—C24—C13	124.09 (14)
C13—C12—H12B	109.0	O2—C24—C13	112.79 (14)
H12A—C12—H12B	107.8	O2—C25—H25A	109.5
C24—C13—C12	113.95 (12)	O2—C25—H25B	109.5
C24—C13—C1	111.64 (12)	H25A—C25—H25B	109.5
C12—C13—C1	107.73 (12)	O2—C25—H25C	109.5
C24—C13—C14	109.32 (12)	H25A—C25—H25C	109.5
C12—C13—C14	110.77 (12)	H25B—C25—H25C	109.5
C1—C13—C14	102.87 (11)	C23—N1—C15	113.43 (13)
C16—C14—C15	115.62 (14)	C23—N1—C1	117.60 (13)
C16—C14—C13	115.10 (12)	C15—N1—C1	103.03 (12)
C15—C14—C13	102.60 (12)	C11—O1—C12	116.89 (11)
C16—C14—H14	107.7	C24—O2—C25	116.50 (15)
			
N1—C1—C2—C11	94.97 (15)	C12—C13—C14—C16	−24.73 (17)
C13—C1—C2—C11	−19.09 (18)	C1—C13—C14—C16	−139.61 (13)
N1—C1—C2—C3	−89.82 (16)	C24—C13—C14—C15	−131.92 (14)
C13—C1—C2—C3	156.12 (13)	C12—C13—C14—C15	101.71 (14)
C11—C2—C3—C4	175.31 (14)	C1—C13—C14—C15	−13.17 (15)
C1—C2—C3—C4	0.0 (2)	C16—C14—C15—N1	108.16 (15)
C11—C2—C3—C8	−3.5 (2)	C13—C14—C15—N1	−17.95 (16)
C1—C2—C3—C8	−178.76 (13)	C15—C14—C16—C21	−25.5 (2)
C8—C3—C4—C5	−1.2 (2)	C13—C14—C16—C21	93.96 (18)
C2—C3—C4—C5	180.00 (15)	C15—C14—C16—C17	154.05 (15)
C3—C4—C5—C6	0.6 (3)	C13—C14—C16—C17	−86.49 (18)
C4—C5—C6—C7	0.0 (3)	C21—C16—C17—C18	0.5 (3)
C5—C6—C7—C8	0.0 (3)	C14—C16—C17—C18	−179.11 (16)
C6—C7—C8—C9	178.23 (18)	C16—C17—C18—C19	−0.3 (3)
C6—C7—C8—C3	−0.7 (3)	C17—C18—C19—C20	−0.2 (3)
C4—C3—C8—C9	−177.71 (15)	C17—C18—C19—C22	179.83 (19)
C2—C3—C8—C9	1.2 (2)	C18—C19—C20—C21	0.7 (3)
C4—C3—C8—C7	1.2 (2)	C22—C19—C20—C21	−179.40 (18)
C2—C3—C8—C7	−179.93 (14)	C19—C20—C21—C16	−0.5 (3)
C7—C8—C9—C10	−177.77 (16)	C17—C16—C21—C20	0.0 (3)
C3—C8—C9—C10	1.1 (2)	C14—C16—C21—C20	179.53 (16)
C8—C9—C10—C11	−1.0 (2)	C12—C13—C24—O3	−155.81 (15)
C3—C2—C11—O1	−173.98 (12)	C1—C13—C24—O3	−33.5 (2)
C1—C2—C11—O1	1.4 (2)	C14—C13—C24—O3	79.66 (18)
C3—C2—C11—C10	3.7 (2)	C12—C13—C24—O2	27.28 (18)
C1—C2—C11—C10	179.09 (13)	C1—C13—C24—O2	149.60 (13)
C9—C10—C11—O1	176.41 (14)	C14—C13—C24—O2	−97.25 (15)
C9—C10—C11—C2	−1.5 (2)	C14—C15—N1—C23	172.67 (14)
O1—C12—C13—C24	66.46 (16)	C14—C15—N1—C1	44.46 (15)
O1—C12—C13—C1	−57.99 (15)	C2—C1—N1—C23	60.86 (17)
O1—C12—C13—C14	−169.79 (11)	C13—C1—N1—C23	−177.89 (13)
N1—C1—C13—C24	156.25 (12)	C2—C1—N1—C15	−173.59 (12)
C2—C1—C13—C24	−80.75 (15)	C13—C1—N1—C15	−52.33 (13)
N1—C1—C13—C12	−77.92 (13)	C2—C11—O1—C12	−13.3 (2)
C2—C1—C13—C12	45.08 (15)	C10—C11—O1—C12	168.89 (12)
N1—C1—C13—C14	39.14 (14)	C13—C12—O1—C11	42.84 (16)
C2—C1—C13—C14	162.14 (11)	O3—C24—O2—C25	−1.3 (3)
C24—C13—C14—C16	101.64 (15)	C13—C24—O2—C25	175.64 (15)

### Hydrogen-bond geometry (Å, °)

**Table:**

*D*—H···*A*	*D*—H	H···*A*	*D*···*A*	*D*—H···*A*
C22—H22A···Cg1^i^	0.96	2.84	3.788 (2)	169

Symmetry codes: (i) −*x*, −*y*+1, −*z*+1.
